# 93 Investigating the minimal requirements for startup procurement by healthcare institutions in Ontario, Canada – CORRIGENDUM

**DOI:** 10.1017/cts.2024.635

**Published:** 2024-10-22

**Authors:** Zoya Aziz Bhatti, Derek Choi, Joseph Ferenbok, Edyta Marcon, Marissa Bird, Juli Smyth, Bibaswan Ghoshal

The above abstract published with an error in the author list. The correct list is shown below.

Zoya Aziz Bhatti, Derek Choi, Joseph Ferenbok, Edyta Marcon, Marissa Bird, Juli Smyth and Bibaswan Ghoshal
